# Supplementary material for: Activation of ILC2s through constitutive IFNγ signaling reduction leads to spontaneous pulmonary fibrosis
Source: Nat Commun. 2023 Dec 14;14:8120. doi: 10.1038/s41467-023-43336-6 (PMC10721793; doi:10.1038/s41467-023-43336-6)
Supplement: Supplementary file 1 — Supplementary Information [file 41467_2023_43336_MOESM1_ESM.pdf]

Supplementary Figures

Supplementary Figure 1

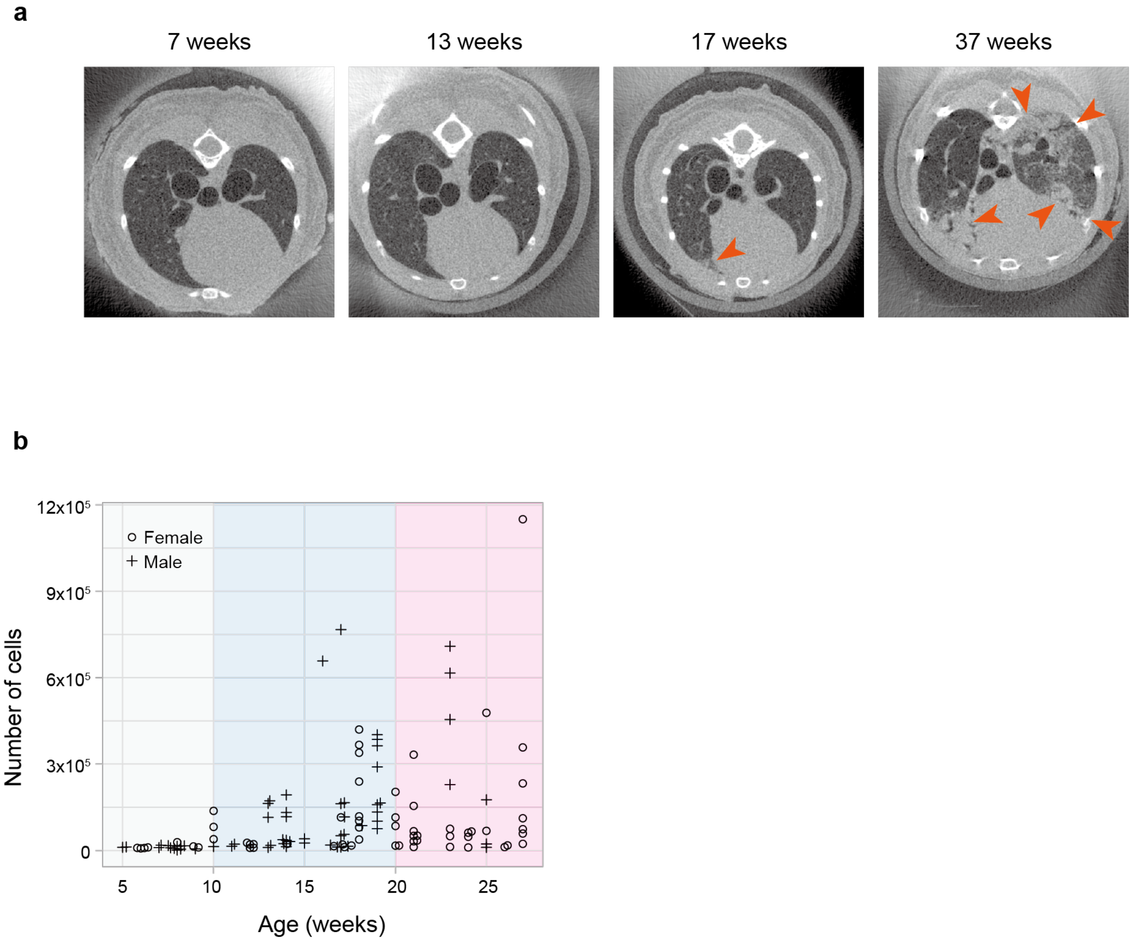

Supplementary Figure 1. *Ifngr1$^{-/-}$Rag2$^{-/-}$* mice spontaneously develop PF

a, Micro-computed tomography (CT) images of *Ifngr1$^{-/-}$Rag2$^{-/-}$* mice at the indicated ages (axial view) (females). The arrows indicate disease areas. **b**, Quantification of the absolute number of whole bronchoalveolar lavage fluid (BALF) cells of *Ifngr1$^{-/-}$Rag2$^{-/-}$* mice of different ages using flow cytometry (n = 119 [60 females, 59 males]). In Fig. 1c, the data were devided by sex and age, and subjected to statistical analyses.

Supplementary Figure 2

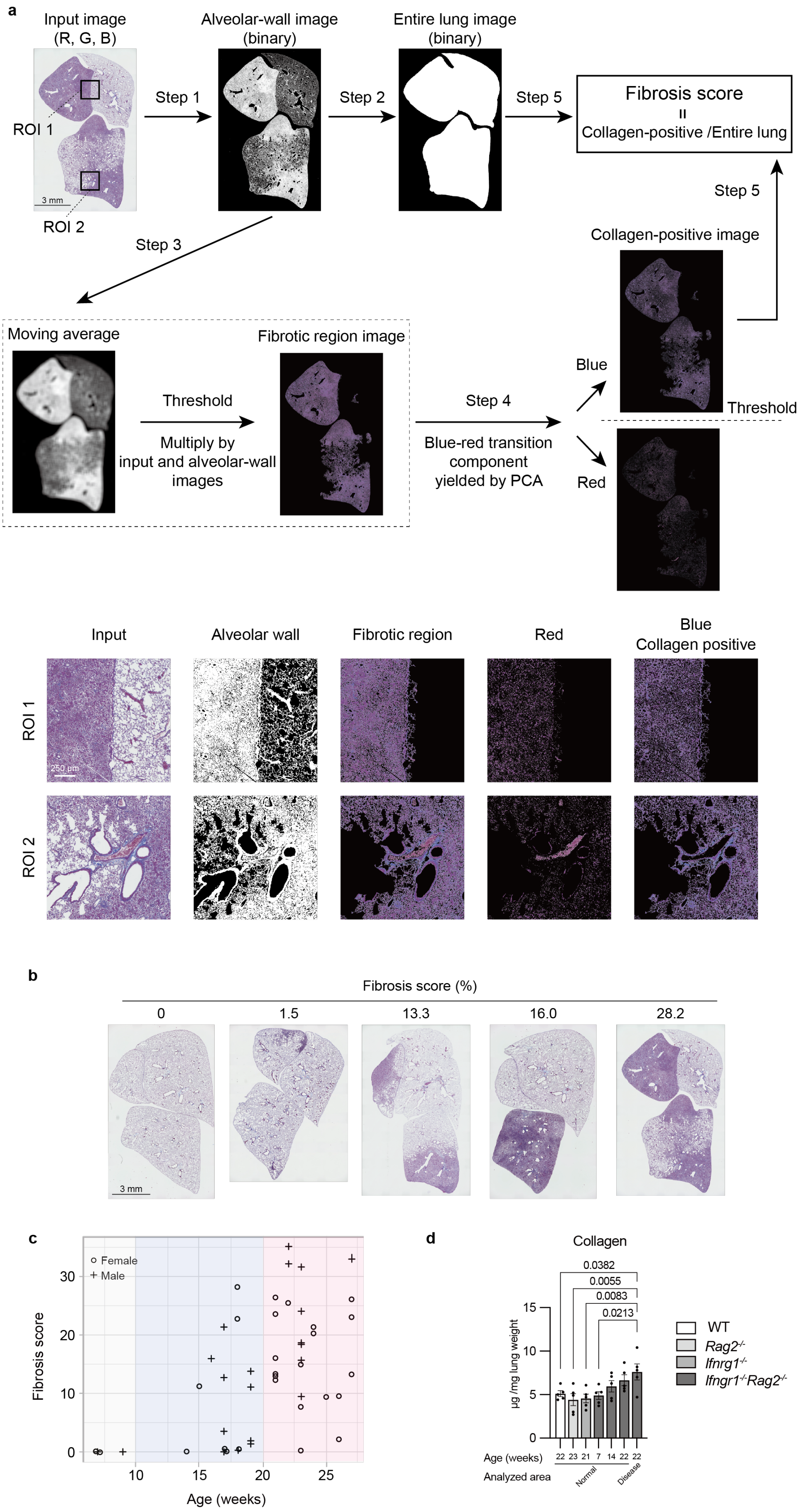

Supplementary Figure 2. Fibrosis score calculation

a, Image-processing pipeline for deriving the Fibrosis score. Details are provided in the Methods and the Code availability sections. The regions of interest (ROIs) indicated by the squares in the upper left input image (ROIs 1 and 2) are enlarged and shown in the lower panels. Scale bar: 3 mm (upper panel) or 250 μm (lower panels). **b,** Examples of the calculated fibrosis scores. Representative Masson's trichrome (MT) -stained images of lung tissue sections from *Ifngr1*$^{-/-}$*Rag2*$^{-/-}$ mice and their corresponding fibrosis scores are shown. Scale bar: 3 mm. **c**, Fibrosis scores of *Ifngr1*$^{-/-}$*Rag2*$^{-/-}$ mice calculated using MT-stained images of their lung tissue sections (n = 48 [27 females, 21 males]). In Fig. 1d, the data were devided by sex and age, and subjected to statistical analyses. **d**, Quantification of the amount of collagen in lung tissue. Specifically, samples of 22-week-old *Ifngr1*$^{-/-}$*Rag2*$^{-/-}$ mice were collected from both the normal and disease areas, whereas all other samples were obtained from the normal area due to the absence of any lesions. The disease area was defined as a visibly distinct white region, as depicted in Fig. 1a. The collagen volume was normalized by the corresponding lung wet weight (n = 5/group; WT and *Ifngr1*$^{-/-}$ mice : females; *Rag2*$^{-/-}$ and *Ifngr1*$^{-/-}$*Rag2*$^{-/-}$ mice: males). Ages of mice were indicated in the figure. Data are representative of at least three independent experiments and are presented as the mean ± s.e.m. For statistical analysis, the following tests were used: **d**, one-way ANOVA with Dunnett's multiple comparisons tests. For **d**, source data are provided as a Source Data file.

Supplementary Figure 3

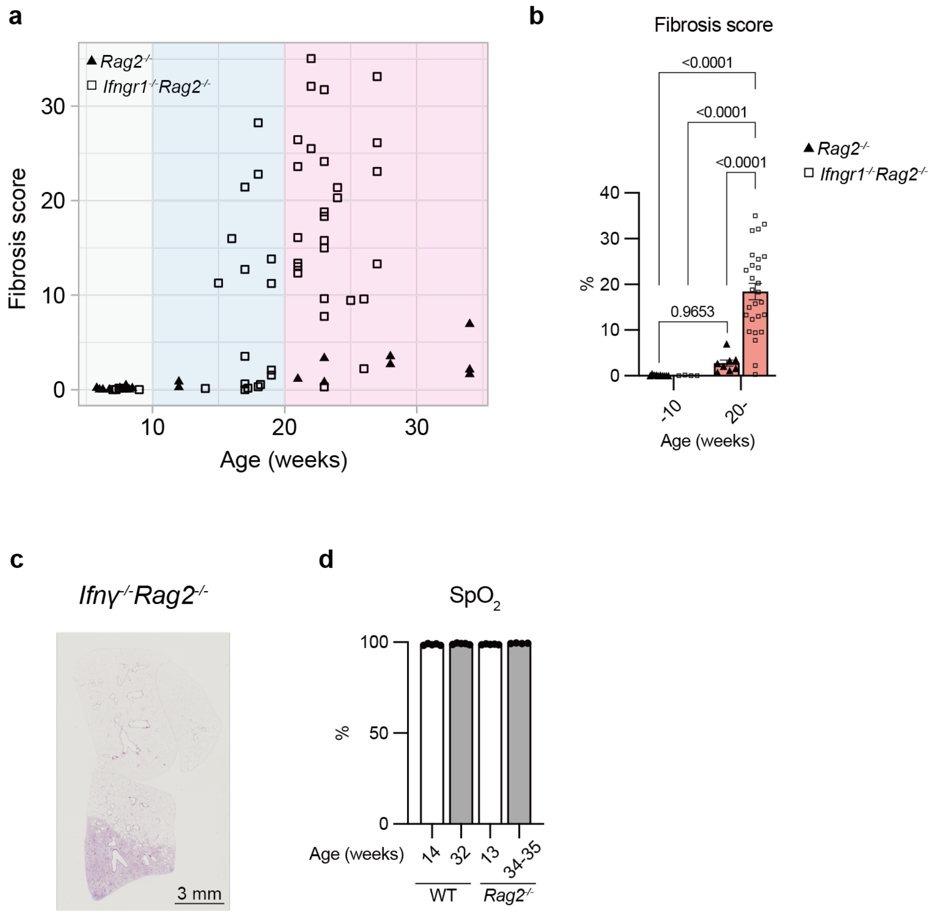

Supplementary Figure 3. Fibrosis scores of *Ifngr1$^{-/-}$Rag2$^{-/-}$* mice and other strains

a and **b**, Fibrosis scores of *Ifngr1$^{-/-}$Rag2$^{-/-}$* and *Rag2$^{-/-}$* mice were calculated using MT-stained images of their lung tissue sections (*Ifngr1$^{-/-}$Rag2$^{-/-}$* mice: n = 48 [27 females and 21 males], *Rag2$^{-/-}$* mice: n = 19 [13 females and 6 males]). The detailed definition of the score is explained in Supplementary Fig. 2. **b,** Statistical analysis of the fibrosis scores. All data in the intact phase (< 10 weeks) and fibrosis phase (> 20 weeks) were used for comparison. **c,** Representative Masson's trichrome (MT)-stained images of lung tissue sections from *Ifng$^{-/-}$Rag2$^{-/-}$* mice (21 weeks; female). Scale bar: 3 mm. **d,** SpO_2 of WT and *Rag2$^{-/-}$* mice of different ages as indicated in the graph (WT and 13 weeks *Rag2$^{-/-}$* mice: n = 5/group; females, 34-35 weeks *Rag2$^{-/-}$* mice: n = 4/group; females). Data, except for **d**, are representative of at least three independent experiments and are presented as the mean ± s.e.m. For statistical analysis, the following tests were used: **b**, two-way ANOVA with Sidak's multiple comparisons tests; **d**, one-way ANOVA with Sidak's multiple comparisons tests. For **b** and **d**, source data are provided as a Source Data file.

Supplementary Figure 4

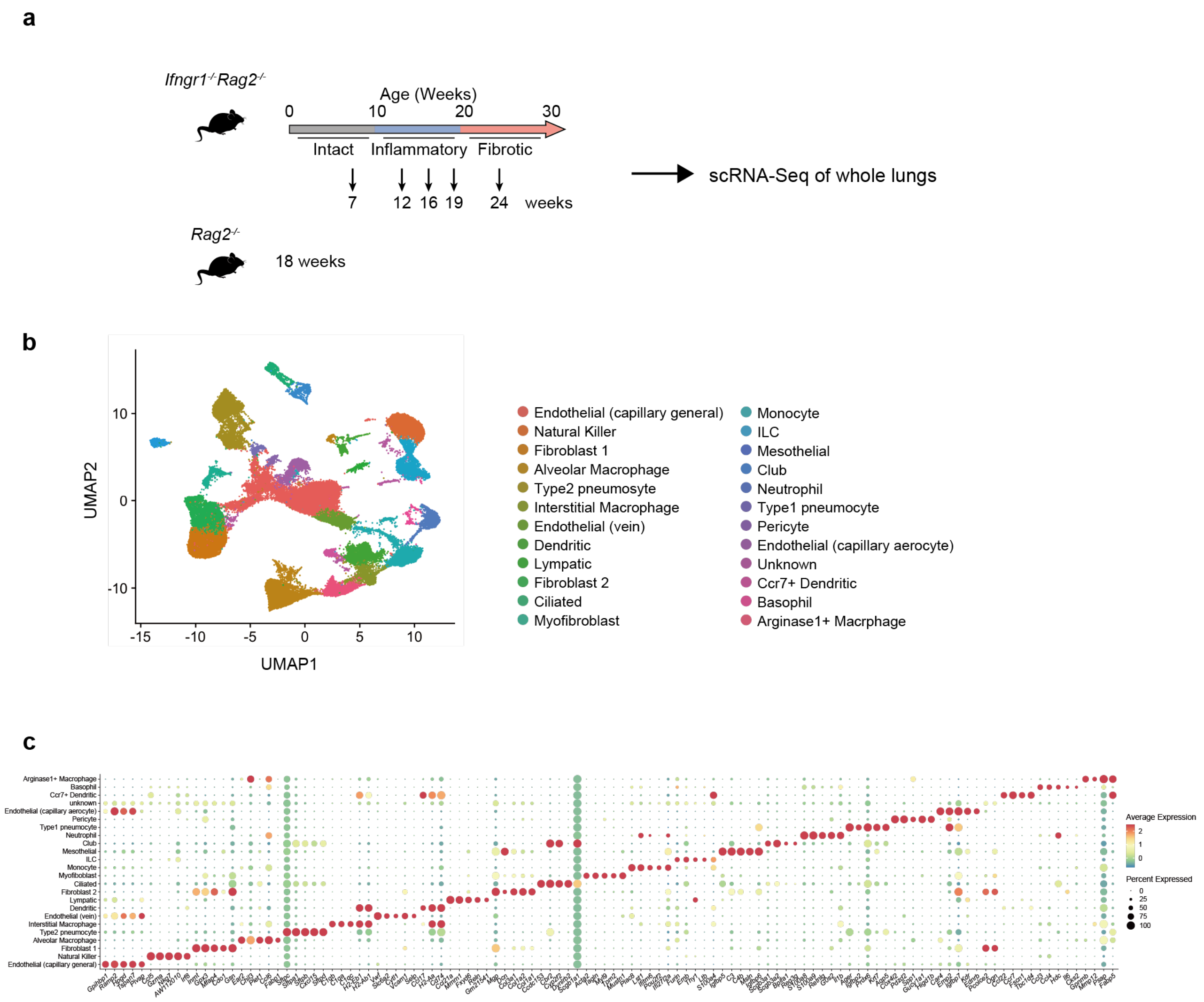

Supplementary Figure 4. scRNA-seq of whole lung cells from *Ifngr1*$^{-/-}$*Rag2*$^{-/-}$ mice of different ages

a, Schematic of the experiment. scRNA-seq of whole lung cells from *Rag2*$^{-/-}$ mice (18 weeks old) and *Ifngr1*$^{-/-}$*Rag2*$^{-/-}$ mice (7, 12, 16, 19, and 24 weeks old) was performed (n = 2/group; females). **b**, Unsupervised clustering of the combined data set was plotted on UMAP and colored according to the identified cell types. **c**, Dot plot of all cell types, showing the top 5 most highly expressed genes in each cell type.

Supplementary Figure 5

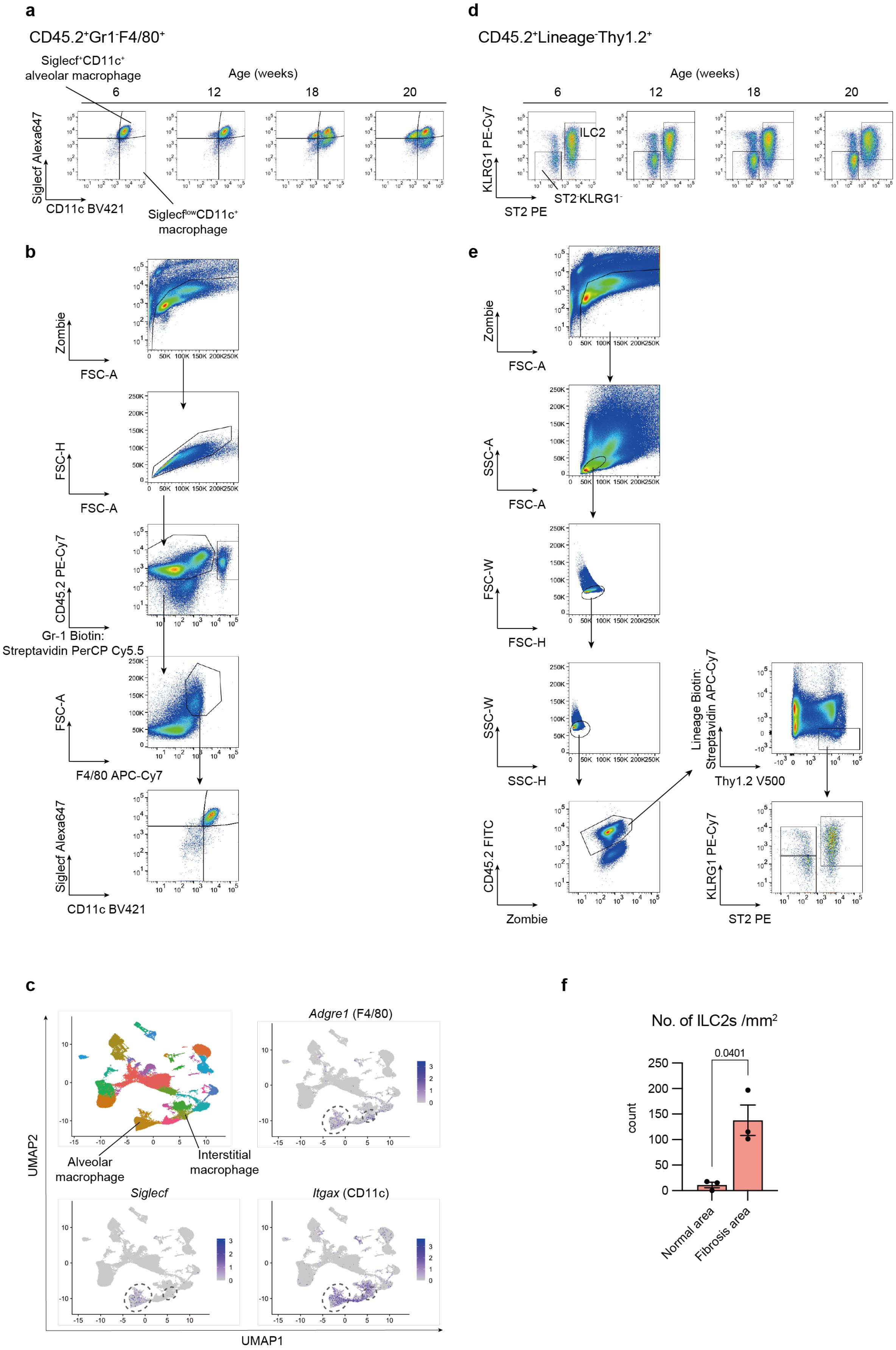

Supplementary Figure 5. Analysis of cellular dynamics during disease progression

a, **b**, **d**, and **e**, Flow cytometry analysis of the lungs of $Ifngr1^{-/-}Rag2^{-/-}$ mice at different ages as indicated in the graphs (6 and 20 weeks: n = 4/group; females, 12 and 18 weeks: n = 5/group; females). **a** and **d**, Representative flow cytometry plots showing the indicated cells in the lungs at each age. Pre-gates are indicated on the plots. **b**, Gating strategy for macrophages (defined as CD45^{+}Gr-1^{-}F4/80^{+}). In Fig. 3g, statistical analyses of the absolute number of defined cells are shown. **e**, Gating strategy for ILC2s (defined as CD45^{+}lineage^{-} [CD3ε, CD4, CD8α, CD11c, FcεRIα, NK1.1, CD19, TER119, F4/80, Ly-6G, and Ly-6C] Thy-1^{+}ST2^{+}KLRG1^{+}) and ST2^{-}KLRG1^{-} cells (defined by CD45^{+}lineage^{-} [CD3ε, CD4, CD8α, CD11c, FcεRIα, NK1.1, CD19, TER119, F4/80, Ly-6G and Ly-6C] Thy-1^{+}ST2^{-}KLRG1^{-}). In Fig. 3h and 3i, statistical analyses of the absolute number of defined cells are shown. **c,** scRNA-seq of whole lung cells from $Rag2^{-/-}$ mice (18 weeks old) and $Ifngr1^{-/-}Rag2^{-/-}$ mice (7, 12, 16, 19, and 24 weeks old) (n = 2/group; females). The upper left panel shows clusters identified as alveolar and interstitial macrophages. Other panels show the expression of the indicated genes. **f,** Quantification of the number of ILC2s per unit area in both normal and fibrosis areas of the lung tissue from $Ifngr1^{-/-}Rag2^{-/-}$ mice in the fibrosis phase, based on fluorescence immunostaining images (n = 3 [1 at 21 weeks, 1 at 24 weeks, 1 at 29 weeks]; 2 males and 1 female). The area where the normal alveolar structure is preserved was defined as the normal area, and the area where the regular alveolar structure is disrupted was defined as the fibrosis area. See Supplementary Fig. 8e for details on the analysis process. For statistical analysis, the following tests were used: **f**, two-tailed paired Student's t-test. For **f**, source data are provided as a Source Data file.

Supplementary Figure 6

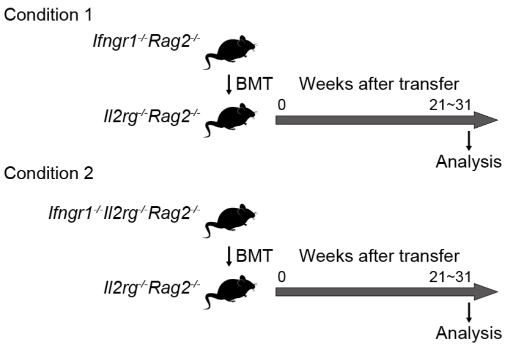

Supplementary Figure 6. Depletion of ILCs from *Ifngr1*$^{-/-}$*Rag2*$^{-/-}$ mice

The details of the bone marrow transfer experiment are explained in Fig. 4c–4e. Schematic of the experiment. Bone marrow cells of either *Ifngr1*$^{-/-}$*Rag2*$^{-/-}$ mice (7 weeks; females) or *Ifngr1*$^{-/-}$*Il2rg*$^{-/-}$ *Rag2*$^{-/-}$ mice (7 weeks; males) were intravenously transferred to *Il2rg*$^{-/-}$*Rag2*$^{-/-}$ mice (12 weeks old at transfer; n = 4/group; females). The mice were sacrificed and analyzed 21–31 weeks after the transfer. BMT, bone marrow transfer.

Supplementary Figure 7

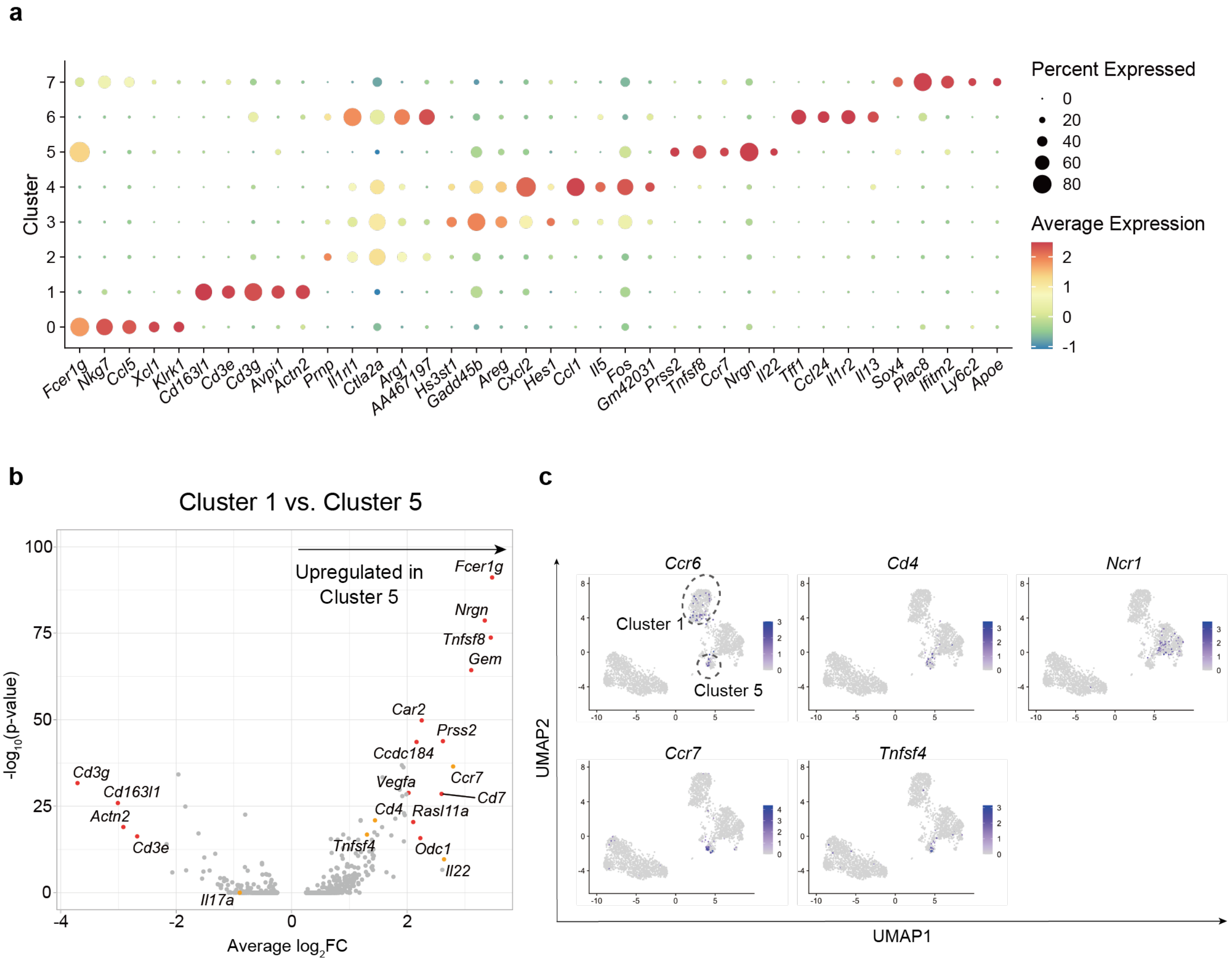

Supplementary Figure 7. scRNA-seq of ILC subsets

a, Dot plot of each ILC sub-cluster, showing the top 5 most highly expressed genes. **b**, Volcano plot of differentially expressed genes (log_2[fold change] > 0.25) between cluster 1 and cluster 5. Upregulated genes (|log_2[fold change]| > 2; $P < 10^{-15}$) are highlighted in red. ILC3-related genes are highlighted in orange. **c**, The expression of ILC3-related genes in ILCs. The dotted circles show cluster 1 and 5. For statistical analysis, the following tests were used: **b**, two-sided Wilcoxon Rank Sum test with the Bonferroni method.

Supplementary Figure 8

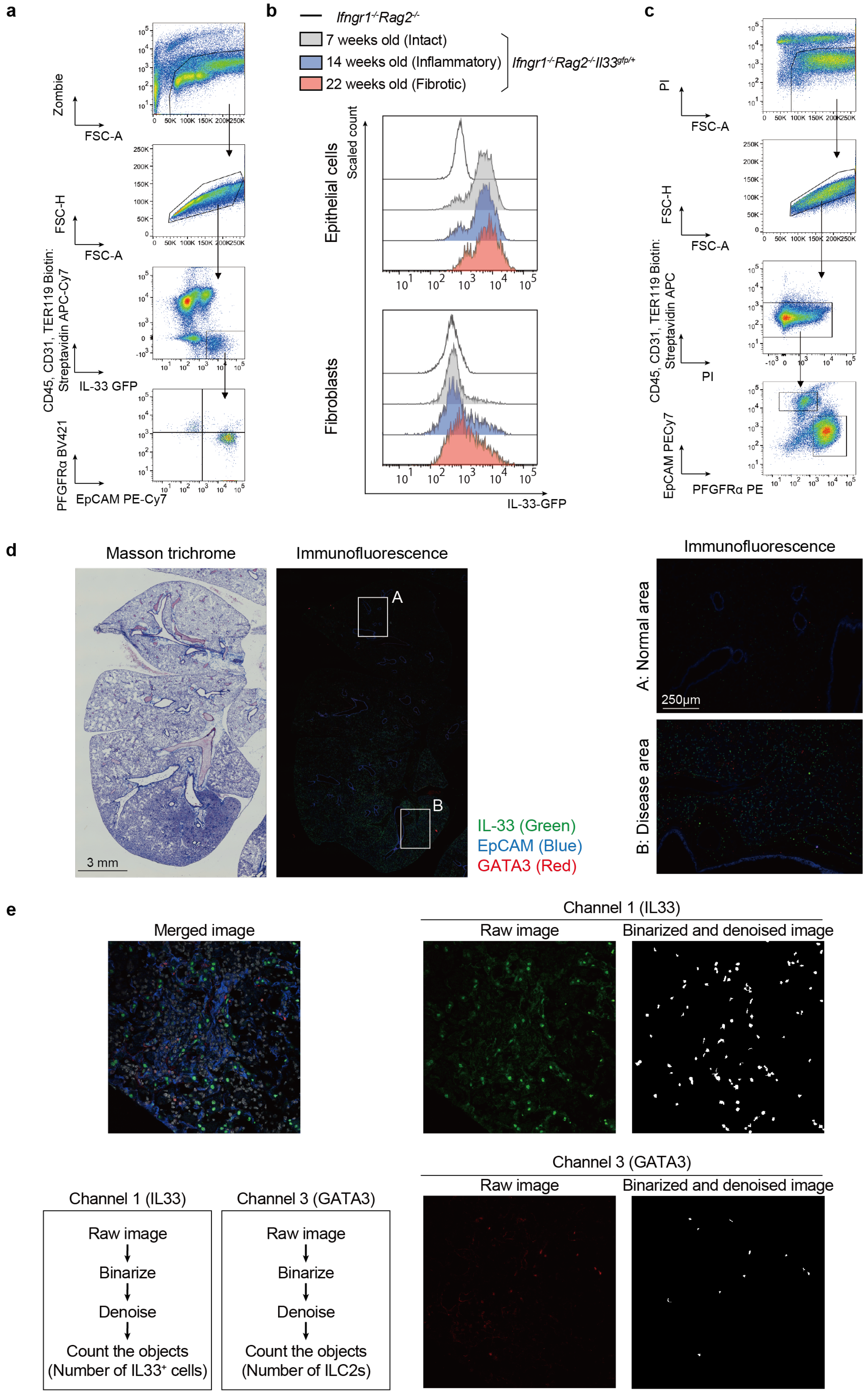

Supplementary Figure 8. Analysis of IL33-expressing cells in the lungs of *Ifngr1*$^{-/-}$*Rag2*$^{-/-}$ mice

a, Gating strategy for identifying GFP-expressing cells in the lungs. Epithelial cells are defined as TER119^{-}CD31^{-}CD45^{-}EpCAM^{+}PDGFRα^{-} while fibroblasts are defined as TER119^{-}CD31^{-}CD45^{-}EpCAM^{-}PDGFRα^{+}. Resulted gates are shown in Fig. 6g. **b**, The expression of IL-33-GFP in epithelial cells (defined as TER119^{-}CD31^{-}CD45^{-}EpCAM^{+}PDGFRα^{-}) and fibroblasts (defined as TER119^{-}CD31^{-}CD45^{-}EpCAM^{-}PDGFRα^{+}) of the indicated mice, measured by flow cytometry (n = 1/group; males). The modes are normalized to the same value. **c**, Gating strategy for sorting of fibroblasts (defined as TER119^{-}CD31^{-}CD45^{-}EpCAM^{-}PDGFRα^{+}) and epithelial cells (defined as TER119^{-}CD31^{-}CD45^{-}EpCAM^{+}PDGFRα^{-}) from the lungs. IL-33-expression of the sorted cells is shown in Fig. 6h. **d**, MT and immunofluorescence staining images of the lungs of *Ifngr1*$^{-/-}$*Rag2*$^{-/-}$ mice (24 weeks; male). The areas enclosed by the squares in the left panel are enlarged and shown in the right panels (A: normal area, and B: disease area). The area where the normal alveolar structure is preserved was defined as the normal area, and the area where the regular alveolar structure is disrupted was defined as the disease area. Green, IL-33: Blue, EpCAM; Red, Gata3. Scale bar: 3 mm (left panels) or 250 μm (right panels). **e**, Quantification of IL33^{+} cells and GATA3^{+} cells (ILC2s) in the immunofluorescence staining images. Images were processed by channel. First, a threshold was set, and the image was binarized. The images were then processed to remove noise (nonspecific staining of small structures), and the number of objects (connected component) per image was counted. Details are provided in the Methods and the Code availability sections. Fig. 6j displays the results of the statistical analysis conducted on the obtained data.

Supplementary Figure 9

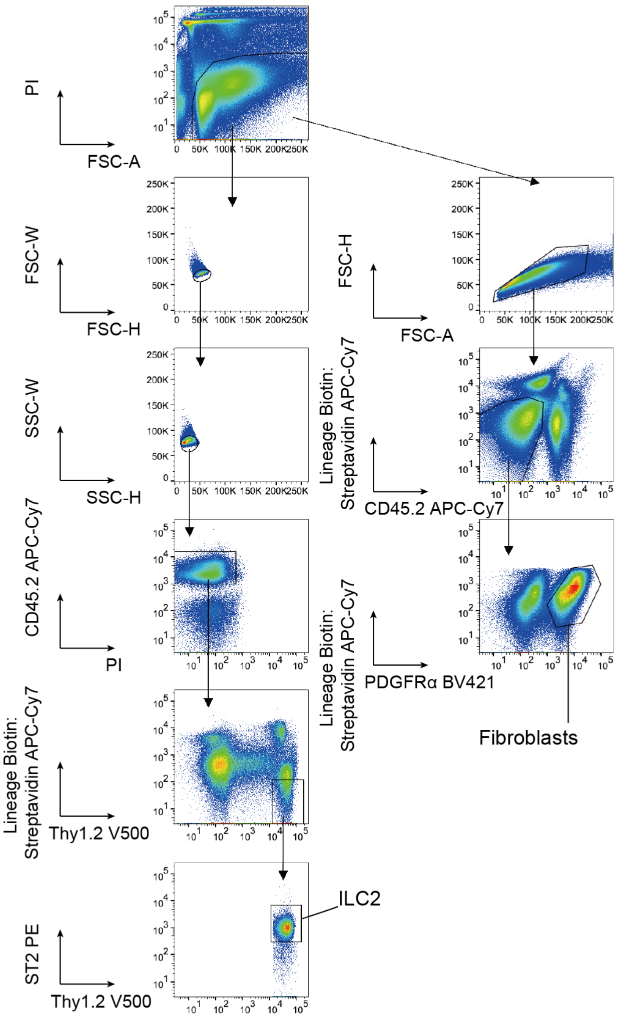

Supplementary Figure 9. Co-culture of ILC2s and fibroblasts

Gating strategy for sorting ILC2s (defined as CD45^{+}lineage^{-} [CD3ε, CD4, CD8α, CD11c, FcεRIα, NK1.1, CD19, TER119, F4/80, Ly-6G, and Ly-6C] Thy-1^{+}ST2^{+}KLRG1^{+}) and fibroblasts (defined as CD45^{-}lineage^{-}CD31^{-}EpCAM^{-}PDGFRα^{+}) from lungs. The sorted cells were used for co-culture analysis shown in Fig. 7.

Supplementary Figure 10

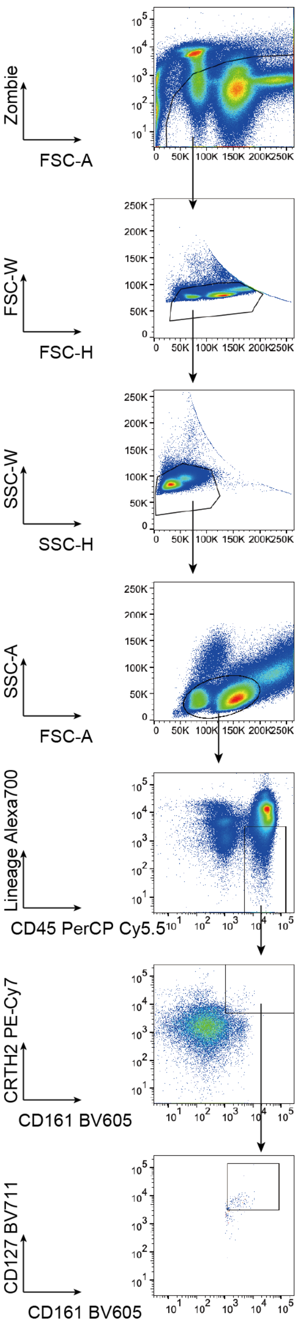

Supplementary Figure 10. RNA-seq of peripheral blood ILC2s from patients with IPF

Gating strategy for sorting ILC2s (defined as $Lineage^-$ [CD3, CD4, CD14, CD16, CD19, and FcεR1α] $CD45^+CD161^+CRTH2^+CD127^+$).The sorted cells were subjected to RNA-seq shown in Fig. 8.
