# Supplementary material for: Activation of ILC2s through constitutive IFNγ signaling reduction leads to spontaneous pulmonary fibrosis
Source: Nat Commun. 2023 Dec 14;14:8120. doi: 10.1038/s41467-023-43336-6 (PMC10721793; doi:10.1038/s41467-023-43336-6)
Supplement: Supplementary file 3 — Description of Additional Supplementary Files [file 41467_2023_43336_MOESM3_ESM.pdf]

Description of Additional Supplementary Files

Supplementary Data 1 – This file contains the concentration of the mAbs.

Supplementary Data 2 – This file contains a description of the characteristics of the study population.
