# Supplementary material for: Activation of ILC2s through constitutive IFNγ signaling reduction leads to spontaneous pulmonary fibrosis
Source: Nat Commun. 2023 Dec 14;14:8120. doi: 10.1038/s41467-023-43336-6 (PMC10721793; doi:10.1038/s41467-023-43336-6)
Supplement: Supplementary file 6 — Reporting Summary [file 41467_2023_43336_MOESM6_ESM.pdf]

nature portfolio

Corresponding author(s): Kazuyo Moro

Last updated by author(s): Oct 20, 2023

Reporting Summary

Nature Portfolio wishes to improve the reproducibility of the work that we publish. This form provides structure for consistency and transparency in reporting. For further information on Nature Portfolio policies, see our Editorial Policies and the Editorial Policy Checklist.

Statistics

For all statistical analyses, confirm that the following items are present in the figure legend, table legend, main text, or Methods section.

n/a	Confirmed	
☐	☒	The exact sample size (n) for each experimental group/condition, given as a discrete number and unit of measurement
☐	☒	A statement on whether measurements were taken from distinct samples or whether the same sample was measured repeatedly
☐	☒	The statistical test(s) used AND whether they are one- or two-sided<br>*Only common tests should be described solely by name; describe more complex techniques in the Methods section.*
☐	☒	A description of all covariates tested
☐	☒	A description of any assumptions or corrections, such as tests of normality and adjustment for multiple comparisons
☐	☒	A full description of the statistical parameters including central tendency (e.g. means) or other basic estimates (e.g. regression coefficient) AND variation (e.g. standard deviation) or associated estimates of uncertainty (e.g. confidence intervals)
☐	☒	For null hypothesis testing, the test statistic (e.g. F, t, r) with confidence intervals, effect sizes, degrees of freedom and P value noted<br>*Give P values as exact values whenever suitable.*
☒	☐	For Bayesian analysis, information on the choice of priors and Markov chain Monte Carlo settings
☒	☐	For hierarchical and complex designs, identification of the appropriate level for tests and full reporting of outcomes
☒	☐	Estimates of effect sizes (e.g. Cohen's d, Pearson's r), indicating how they were calculated

Our web collection on statistics for biologists contains articles on many of the points above.

Software and code

Policy information about availability of computer code

Data collection	Measurement of SP-D, IL-33, and eluted Sirius red: Absorbance was measured with iMark (Bio-Rad, Hercules, CA, USA).<br><br>Micro-CT images: The lungs were scanned by ScanXmate-RX (Comscantechno, Kanagawa, Japan).<br><br>Respiratory function: Static compliance was measured by FinePoint Resistance & Compliance (Primetech, Tokyo, Japan).<br><br>Oxygen saturation: Oxygen saturation was measured by MouseOx PLUS (Primetech).<br><br>Flow cytometry analysis: Data were taken by FACSAria IIIu (BD FACS Diva Software Version 9.0.1) or FACSCanto II flow cytometer (BD FACS Diva Software Version 8.0.2) (BD Biosciences, Franklin Lakes, NJ, USA).<br><br>Real-time quantitative PCR: The amount of cDNA was measured by ABI 7500 Real-Time PCR System (Thermo Fisher Scientific, Waltham, MA, USA).<br><br>scRNA-seq: Single-cell encapsulation and library construction were performed using a Chromium Controller with a Chromium Single-Cell 3’ v2 Reagents Kit (10x Genomics, Pleasanton, CA, USA), following the manufacturer’s instructions. Libraries were sequenced using a NovaSeq instrument (Illumina, San Diego, CA, USA).<br><br>Bulk RNA-seq: Library construction was performed using a NEBNext Single-Cell/Low Input RNA Library Prep Kit for Illumina (#E6420S, New England Biolabs Japan Inc., Tokyo, Japan), following the manufacturer’s instructions. One sample from a patient with IPF was omitted before

	sequencing because of the low RNA quality. The libraries were sequenced using a HiSeq X™ instrument (Illumina).<br><br>Image analysis: The images were taken by SZX7 (OLYMPUS, Tokyo, Japan), BZ-X700 (KEYENCE, Osaka, Japan), or TCS SP8 (Leica Japan, Tokyo, Japan).
Data analysis	Flow-cytometry analysis: Data were analysed by FlowJo software (v10.8.1) (BD Biosciences).<br><br>Single cell RNA-sequence: NovaSeq Control Software v1.4.0 (Illumina) and Real Time Analysis (RTA) v3.3.3 (Illumina) were used for basecalling. Basecalls were converted to fastq files by using bcl2fastq2 v2.20 software (Illumina). Cell Ranger v3.1.0 (10x Genomics) was used to map sequenced reads to mm10 whole genome. Downstream analysis was carried out using R software (v4.1.3), Seurat package (v4.3.0), and ggplot2 package (v3.4.0).<br><br>Bulk RNA-sequencing: Basecalls were converted to fastq files by using blc2fastq2 package of illumina software. Quality control was performed using fastp (v0.21.0). Gene counts were obtained by aligning reads to the hg38 genome using HISAT2(v2.2.1) followed by processing with samtools (v1.12) and the Rsubread package (v2.4.3) of R software (v4.1.3). Downstream analysis was carried out using DESeq2 package (v1.30.1), clusterProfiler package (v3.14.3), GenomicRanges (v1.42.0) and pheatmap package (v1.0.12).<br><br>Image analysis: Data were analysed by MATLAB (R2022a).<br><br>All statistics were performed by GraphPad Prism (v9.4.0) (GraphPad, Boston, MA, USA).

For manuscripts utilizing custom algorithms or software that are central to the research but not yet described in published literature, software must be made available to editors and reviewers. We strongly encourage code deposition in a community repository (e.g. GitHub). See the Nature Portfolio guidelines for submitting code & software for further information.

Data

Policy information about availability of data

All manuscripts must include a data availability statement. This statement should provide the following information, where applicable:

- Accession codes, unique identifiers, or web links for publicly available datasets
- A description of any restrictions on data availability
- For clinical datasets or third party data, please ensure that the statement adheres to our policy

The scRNA-seq data of ILC2s from lungs of Ifngr1-/-Rag2-/- mice generated in this study have been deposited in the NCBI GEO database under accession code GSE164220
[https://www.ncbi.nlm.nih.gov/geo/query/acc.cgi?acc=GSE164220].
The RNA-seq data of ILC2s from human serum have been deposited in the NCBI GEO database under accession code GSE194244
[https://www.ncbi.nlm.nih.gov/geo/query/acc.cgi?acc=GSE194244].
Source data are provided with this paper.

Mouse genome GRCm38/mm10 was used as a reference for scRNA-seq analysis.
Human genome GRCh38/hg38 was used as a reference for bulk RNA-seq analysis.

Codes for image analysis used in this study have been deposited in the Zendo open data repository under a following DOI [DOI: 10.5281/zenodo.10016650].
Other codes are available from authors upon reasonable request.

Research involving human participants, their data, or biological material

Policy information about studies with human participants or human data. See also policy information about sex, gender (identity/presentation), and sexual orientation and race, ethnicity and racism.

Reporting on sex and gender	Sex and gender data were indicated in Extended Data Table.
Reporting on race, ethnicity, or other socially relevant groupings	Asians were recruited for this study.
Population characteristics	Age and other profiles were indicated in Extended Data Table.
Recruitment	IPF whole blood was obtained from 20 consecutive patients with IPF at Tokai University Hospital. IPF was diagnosed based on a multidisciplinary discussion by two pulmonologists and a radiologist using the patients' clinical history, physical examination, laboratory test results, and radiographic data from high-resolution computed tomography. Patients taking oral corticosteroids > 10 mg prednisolone/day or those with concomitant allergic diseases such as asthma and eczema requiring pharmacological treatments were excluded.
Ethics oversight	Whole blood was obtained from 12 healthy adult volunteers (normal controls) from the Yokohama Minoru Clinic (Kanagawa, Japan) under a protocol approved by the Ethics Committee at the RIKEN Center for Integrative Medical Sciences (approval number: H29-12[5]), and Astellas Pharma Inc. (approval number: 000181). IPF whole blood was obtained from 20 individuals with IPF from Tokai University Hospital (Kanagawa, Japan) under a protocol approved by the Ethics Committee at the RIKEN Center for Integrative Medical Sciences (approval number: H29-12[5]), the Institutional Review Board for Clinical Research, Tokai University (approval number: 17R-157), and Astellas Pharma Inc (approval number: 000181). All the healthy volunteers and patients with IPF provided written informed consent. All methods included in this study were conducted in accordance

with relevant guidelines and regulations.

Note that full information on the approval of the study protocol must also be provided in the manuscript.

Field-specific reporting

Please select the one below that is the best fit for your research. If you are not sure, read the appropriate sections before making your selection.

☒ Life sciences ☐ Behavioural & social sciences ☐ Ecological, evolutionary & environmental sciences

For a reference copy of the document with all sections, see nature.com/documents/nr-reporting-summary-flat.pdf

Life sciences study design

All studies must disclose on these points even when the disclosure is negative.

Sample size	No statistical methods were used to determine sample size. Sample size was determined based on previous experience from our group (PMID: 26595888, 29728440) and publications in the field (PMID: 33674322).<br><br>We utilized 3-10 mice/group in this study.<br>In in vitro experiments involving co-culturing of fibroblasts and ILC2s, cells sorted from 10 mice were combined and seeded into four separate wells. This experiment was repeated multiple times, consistently yielding similar results in each iteration.
Data exclusions	In the bulk RNA-sequening analysis of ILC2s of 12 healthy controls and 19 IPF patients, one sample of patients with IPF was omitted before sequencing because of the low quality of RNA.
Replication	All experiments, except for Extended Data Fig. 1a, Fig. 2c, Extended Data Fig. 3d and Extended Data Fig. 5f, were repeated several times and gave reproducible results, as indicated in each figure legend.<br><br>Extended Data Fig. 1a (Micro-CT images of lungs): The lungs were scanned by ScanXmate-RX (Comscantechno). Since this equipment was not available in the research institute, external equipment was used. Since this particular equipment was not accessible within the research institute, external equipment was employed. Consequently, due to limited availability of external equipment, the experiment could only be conducted once.<br><br>Fig. 2c (Respiratory function test): Static compliance was measured by FinePointe™ Resistance & Compliance (Primetech). ince this equipment was not available in the research institute, external equipment was used. Since this particular equipment was not accessible within the research institute, demonstration equipment was employed. Consequently, due to limited availability of demonstration equipment, the experiment could only be conducted once.
Randomization	All mice were randomly assigned to the control or experimental groups.
Blinding	The investigators were not blinded to group allocation during data collection and analysis. It is not possible because the number of investigators were limited and the investigators had to conduct experiments in addition to data collection and analysis.

Reporting for specific materials, systems and methods

We require information from authors about some types of materials, experimental systems and methods used in many studies. Here, indicate whether each material, system or method listed is relevant to your study. If you are not sure if a list item applies to your research, read the appropriate section before selecting a response.

Materials & experimental systems

n/a	Involved in the study	
☐	☒	Antibodies
☒	☐	Eukaryotic cell lines
☒	☐	Palaeontology and archaeology
☐	☒	Animals and other organisms
☒	☐	Clinical data
☒	☐	Dual use research of concern
☒	☐	Plants

Methods

n/a	Involved in the study	
☒	☐	ChIP-seq
☐	☒	Flow cytometry
☒	☐	MRI-based neuroimaging

Antibodies

Antibodies used	Antibodies used are described below.<br>(Antigen, Conjugate, Clone, Company, Catalog number)<br><br>[Flow cytometry]

anti-mouse:
1. CD11c, PECy7, HL3, BD, 558079
2. CD11c, BV421, HL3, BD, 562782
3. CD11c , Biotin, HL3 ,BD, 553800
4. CD140a, BV421, APA5, Biolegend, 135923
5. CD140a, PE, APA5, Biolegend, 135906
6. CD16/CD32, -, 2.4G2, Hybridoma, -
7. CD19 , Biotin, 1D3, BD, 553784
8. CD31, Biotin, 390, Biolegend, 102404
9. CD3ε, Biotin, 145-2C11, BD, 553060
10. CD4, Biotin, GK1. 5, BD, 553728
11. CD45, Biotin, 30-F11, Biolegend, 103104
12. CD45.2, FITC, 104, BD, 553772
13. CD45.2, PECy7, 104, BD, 560696
14. CD45.2, BV421, 104, BD, 562895
15. CD45.2, APCCy7, 104, BD, 560694
16. CD8α, Biotin, 53-6.7, Hybridoma, -
17. EpCAM, PECy7, G8.8, Biolegend, 118216
18. EpCAM, Biotin, 78.8, Biolegend, 118204
19. F4/80, Biotin, BM8, eBioscience, 13-4801-85
20. F4/80, APCCy7, BM8, Biolegend, 123118
21. FceRIα, Biotin, MAR-1, eBioscience, 13-5898-85
22. KLRG1, PECy7, 2F1, eBioscience, 25-5893-82
23. KLRG1, PeCPCy5.5, 2F1, BD, 583595
24. Ly-6G and Ly-6C (Gr-1), Biotin, RB6-8C5, BD, 553125
25. NK1.1, Biotin, PK136, Hybridoma, -
26. SiglecF, Alexa647, E50-2440, BD, 562680
27. T1/ST2, PE, U29-93, BD, 566311
28. TER119, Biotin, TER119, Hybridoma, -
29. Thy1.2, V500, 53-2.1, BD, 561616

anti-human:
30. CD45, PerCP/Cy5.5, HI30, Biolegend, 304028
31. CRTH2 , PE/Cy7, BM16, Biolegend, 350118
32. CD3, Alexa 700, UCHT1, Biolegend, 300424
33. CD4, Alexa 700, RPA-T4 , Biolegend, 300526
34. CD14, Alexa 700, HCD14, Biolegend, 325614
35. CD16, Alexa 700, 3G8 , Biolegend, 302026
36. CD19, Alexa 700, HIB19, Biolegend, 302226
37. FceR1a, Alexa 700, AER-37, Biolegend, 334630
38. CD161, BV605, HP-3G10 Biolegend, 339916
39. CD127 , BV711, hIL-7R-M21BD, BD, 563165

Other:
40. Streptavidin, APCCy7, -, BD, 554063
41. Streptavidin, APC, -, BD, 554067
42. Streptavidin, PerCPCy5.5, -, BD, 551419

[Immunofluorescence staining]
anti-mouse:
43. IL-33, purified, -, R&D, AF3626
44. GATA3, Alexa647, L50-823, BD, 560078
45. PFGFRα, purified, EPR22059-27, abcam, ab203491
46. EpCAM(CD326), biotin, G8.8, Biolegend, 118204

Validation

The following antibodies were purified from hybridoma culture supernatant in our laboratory. Validation of these antibodies was carried out by comparison with commercially available antibodies.

6. CD16/CD32, -, 2.4G2, Hybridoma, -
16. CD8α, Biotin, 53-6.7, Hybridoma, -
25. NK1.1, Biotin, PK136, Hybridoma, -
28. TER119, Biotin, TER119, Hybridoma, -

All other antubidies were purchased and only used, according to the manufacturer's instructions.
The validation procedures were described on the following sites of the manufactures.

1. CD11c, PECy7, HL3, BD, 558079 (https://www.bdbiosciences.com/en-us/products/reagents/flow-cytometry-reagents/research-reagents/single-color-antibodies-ruo/pe-cy-7-hamster-anti-mouse-cd11c.558079)
2. CD11c, BV421, HL3, BD, 562782 (https://www.bdbiosciences.com/en-us/products/reagents/flow-cytometry-reagents/research-

reagents/single-color-antibodies-ruo/bv421-hamster-anti-mouse-cd11c.562782)
3. CD11c , Biotin, HL3 ,BD, 553800 (https://www.bdbiosciences.com/en-us/products/reagents/flow-cytometry-reagents/research-reagents/single-color-antibodies-ruo/biotin-hamster-anti-mouse-cd11c.553800)
4. CD140a, BV421, APA5, Biolegend, 135923 (https://www.biolegend.com/ja-jp/products/brilliant-violet-421-anti-mouse-cd140a-antibody-17921?GroupID=ImportedGROUP1)
5. CD140a, PE, APA5, Biolegend, 135906 (https://www.biolegend.com/en-us/products/pe-anti-mouse-cd140a-antibody-6253?GroupID=BLG8105)
7. CD19 , Biotin, 1D3, BD, 553784 (https://www.bdbiosciences.com/en-us/products/reagents/flow-cytometry-reagents/research-reagents/single-color-antibodies-ruo/biotin-rat-anti-mouse-cd19.553784)
8. CD31, Biotin, 390, Biolegend, 102404 (https://www.biolegend.com/ja-jp/products/biotin-anti-mouse-cd31-antibody-119)
9. CD3ε, Biotin, 145-2C11, BD, 553060 (https://www.bdbiosciences.com/en-us/products/reagents/flow-cytometry-reagents/research-reagents/single-color-antibodies-ruo/biotin-hamster-anti-mouse-cd3e.553060)
10. CD4, Biotin, GK1. 5, BD, 553728 (https://www.bdbiosciences.com/en-us/products/reagents/flow-cytometry-reagents/research-reagents/single-color-antibodies-ruo/biotin-rat-anti-mouse-cd4.553728)
11. CD45, Biotin, 30-F11, Biolegend, 103104 (https://www.biolegend.com/ja-jp/products/biotin-anti-mouse-cd45-antibody-98?GroupID=BLG1932)
12. CD45.2, FITC, 104, BD, 553772 (https://www.bdbiosciences.com/en-us/products/reagents/flow-cytometry-reagents/research-reagents/single-color-antibodies-ruo/fitc-mouse-anti-mouse-cd45-2.553772)
13. CD45.2, PECy7, 104, BD, 560696 (https://www.bdbiosciences.com/en-us/products/reagents/flow-cytometry-reagents/research-reagents/single-color-antibodies-ruo/pe-cy-7-mouse-anti-mouse-cd45-2.560696)
14. CD45.2, BV421, 104, BD, 562895 (https://www.bdbiosciences.com/en-us/products/reagents/flow-cytometry-reagents/research-reagents/single-color-antibodies-ruo/bv421-mouse-anti-mouse-cd45-2.562895)
15. CD45.2, APCCy7, 104, BD, 560694 (https://www.bdbiosciences.com/en-us/products/reagents/flow-cytometry-reagents/research-reagents/single-color-antibodies-ruo/apc-cy-7-mouse-anti-mouse-cd45-2.560694)
17. EpCAM, PECy7, G8.8, Biolegend, 118216 (https://www.biolegend.com/en-us/products/pe-cyanine7-anti-mouse-cd326-ep-cam-antibody-5303?GroupID=BLG6455)
18. EpCAM, Biotin, 78.8, Biolegend, 118204 (https://www.biolegend.com/ja-jp/products/biotin-anti-mouse-cd326-ep-cam-antibody-4725?GroupID=BLG6455)
19. F4/80, Biotin, BM8, eBioscience, 13-4801-85 (https://www.thermofisher.com/antibody/product/F4-80-Antibody-clone-BM8-Monoclonal/13-4801-82)
20. F4/80, APCCy7, BM8, Biolegend, 123118 (https://www.biolegend.com/ja-jp/products/apc-cyanine7-anti-mouse-f4-80-antibody-4072?GroupID=BLG5319)
21. FceRIα, Biotin, MAR-1, eBioscience, 13-5898-85 (https://www.thermofisher.com/antibody/product/FceR1-alpha-Antibody-clone-MAR-1-Monoclonal/13-5898-82)
22. KLRG1, PECy7, 2F1, eBioscience, 25-5893-82 (https://www.thermofisher.com/antibody/product/KLRG1-Antibody-clone-2F1-Monoclonal/25-5893-82)
23. KLRG1, PeCPCy5.5, 2F1, BD, 583595 (https://www.bdbiosciences.com/en-us/products/reagents/flow-cytometry-reagents/research-reagents/single-color-antibodies-ruo/percp-cy-5-5-hamster-anti-mouse-klrg1.563595)
24. Ly-6G and Ly-6C (Gr-1), Biotin, RB6-8C5, BD, 553125 (https://www.bdbiosciences.com/en-us/products/reagents/cell-preparation-separation-reagents/biotin-rat-anti-mouse-ly-6g-and-ly-6c.553125)
26. SiglecF, Alexa647, E50-2440, BD, 562680 (https://www.bdbiosciences.com/en-us/products/reagents/flow-cytometry-reagents/research-reagents/single-color-antibodies-ruo/alexa-fluor-647-rat-anti-mouse-siglec-f.562680)
27. T1/ST2, PE, U29-93, BD, 566311 (https://www.bdbiosciences.com/en-us/products/reagents/flow-cytometry-reagents/research-reagents/single-color-antibodies-ruo/pe-rat-anti-mouse-il-33r-st2.566311)
29. Thy1.2, V500, 53-2.1, BD, 561616 (https://www.bdbiosciences.com/en-us/products/reagents/flow-cytometry-reagents/research-reagents/single-color-antibodies-ruo/v500-rat-anti-mouse-cd90-2.561616)
30. CD45, PerCP/Cy5.5, HI30, Biolegend, 304028 (https://www.biolegend.com/ja-jp/products/percp-cyanine5-5-anti-human-cd45-antibody-4240?GroupID=BLG5926)
31. CRTH2 , PE/Cy7, BM16, Biolegend, 350118 (https://www.biolegend.com/ja-jp/search-results/pe-cyanine7-anti-human-cd294-crth2-antibody-8815?GroupID=BLG9051)
32. CD3, Alexa 700, UCHT1, Biolegend, 300424 (https://www.biolegend.com/ja-jp/search-results/alexa-fluor-700-anti-human-cd3-antibody-3394)
33. CD4, Alexa 700, RPA-T4 , Biolegend, 300526 (https://www.biolegend.com/ja-jp/cell-health/alexa-fluor-700-anti-human-cd4-antibody-3395)
34. CD14, Alexa 700, HCD14, Biolegend, 325614 (https://www.biolegend.com/ja-jp/products/alexa-fluor-700-anti-human-cd14-antibody-3956)
35. CD16, Alexa 700, 3G8 , Biolegend, 302026 (https://www.biolegend.com/ja-jp/products/alexa-fluor-700-anti-human-cd16-antibody-3398)
36. CD19, Alexa 700, HIB19, Biolegend, 302226 (https://www.biolegend.com/ja-jp/products/alexa-fluor-700-anti-human-cd19-antibody-3399)
37. FceR1a, Alexa 700, AER-37, Biolegend, 334630 (https://www.biolegend.com/ja-jp/products/alexa-fluor-700-anti-human-fcepsilonrialpha-antibody-12981?GroupID=BLG6051)
38. CD161, BV605, HP-3G10 Biolegend, 339916 (https://www.biolegend.com/ja-jp/products/brilliant-violet-605-anti-human-cd161-antibody-7672?GroupID=BLG10204)
39. CD127 , BV711, hIL-7R-M21BD, BD, 563165 (https://www.bdbiosciences.com/en-us/products/reagents/flow-cytometry-reagents/research-reagents/single-color-antibodies-ruo/bv711-mouse-anti-human-cd127.563165)
40. Streptavidin, APCCy7, -, BD, 554063 (https://www.bdbiosciences.com/en-tw/products/reagents/flow-cytometry-reagents/research-reagents/single-color-antibodies-ruo/streptavidin-apc-cy-7.554063)
41. Streptavidin, APC, -, BD, 554067 (https://www.bdbiosciences.com/en-us/products/reagents/flow-cytometry-reagents/research-reagents/single-color-antibodies-ruo/apc-streptavidin.554067)
42. Streptavidin, PerCPCy5.5, -, BD, 551419 (https://www.bdbiosciences.com/en-us/products/reagents/flow-cytometry-reagents/research-reagents/single-color-antibodies-ruo/percp-cy-5-5-streptavidin.551419)

43. IL-33, purified, -, R&D, AF3626 (https://www.rndsystems.com/products/mouse-il-33-antibody_af3626)
44. GATA3, Alexa647, L50-823, BD, 560078 (https://www.bdbiosciences.com/en-us/products/reagents/microscopy-imaging-reagents/immunofluorescence-reagents/alexa-fluor-647-mouse-anti-gata3.560078)
45. PFGFRα, purified, EPR22059-27, abcam, ab203491 (https://www.abcam.com/products/primary-antibodies/pdgfr-alpha-antibody-epr22059-270-ab203491.html)
46. EpCAM(CD326), biotin, G8.8, Biolegend, 118204 (https://www.biolegend.com/ja-jp/products/biotin-anti-mouse-cd326-ep-cam-antibody-4725?GroupID=BLG6455)

Animals and other research organisms

Policy information about studies involving animals; ARRIVE guidelines recommended for reporting animal research, and Sex and Gender in Research

Laboratory animals	WT C57BL/6N mice were purchased from Charles River Laboratories Japan (Kanagawa, Japan) or CLEA (Tokyo, Japan). B6- Rag2-/- (stock no. RAGN12) and Il2rg-/-Rag2-/- mice (stock no. 4111) were purchased from Taconic Bioscience Japan (Tokyo, Japan). Ifngr1-/- mice (stock no. 003288) were purchased from Jackson Laboratory and crossed with Rag2-/- mice to generate Ifngr1-/-Rag2-/- mice. Ifng-/- mice were provided by Yoichiro Iwakura (Tokyo University of Science, Chiba, Japan), Il33gfp/gfp mice were provided by Susumu Nakae (Hiroshima University, Hiroshima, Japan), and Rorcgfp/gfp mice were provided by Sidonia Fagarasan (RIKEN, Yokohama, Japan). Ifng-/- mice were crossed with Rag2-/- mice to generate Ifng-/-Rag2-/- mice. Rorcgfp/gfp mice were crossed with Rag2-/- mice to generate Rorcgfp/gfpRag2-/- mice. Ifngr1-/-Rag2-/- mice were crossed with Il2rg-/-Rag2-/-, Rorcgfp/gfpRag2-/-, or Il33gfp/gfp mice to generate Ifngr1-/-Il2rg-/-Rag2-/-, Ifngr1-/-Rorcgfp/gfpRag2-/-, Ifngr1-/-Rag2-/-Il33gfp/+, and Ifngr1-/-Rag2-/-Il33gfp/gfp mice.
Wild animals	Wild animals were not used in this study.
Reporting on sex	We initially verified the development of fibrosis in both male and female mice (Fig. 1c and d). Consequently, both sexes were included in all subsequent experiments. Further information regarding the sex of the mice in each dataset is provided in the figure legends and the Source data.
Field-collected samples	Field-collected samples were not used in this study.
Ethics oversight	All mice used in this study had a C57BL/6 background and were maintained under specific pathogen-free conditions in an animal facility at the RIKEN Center for Integrative Medical Sciences (Kanagawa, Japan). Mice were housed in an environment with a 12-hour light/dark cycle, maintaining an ambient temperature between 21°C and 25°C, and humidity levels controlled at 40-60%. All experiments were approved by the Animal Care and Use Committee of RIKEN and performed in accordance with the institutional guidelines. All mice used in this study were euthanized under anesthesia.

Note that full information on the approval of the study protocol must also be provided in the manuscript.

Plants

Seed stocks	*Report on the source of all seed stocks or other plant material used. If applicable, state the seed stock centre and catalogue number. If plant specimens were collected from the field, describe the collection location, date and sampling procedures.*
Novel plant genotypes	*Describe the methods by which all novel plant genotypes were produced. This includes those generated by transgenic approaches, gene editing, chemical/radiation-based mutagenesis and hybridization. For transgenic lines, describe the transformation method, the number of independent lines analyzed and the generation upon which experiments were performed. For gene-edited lines, describe the editor used, the endogenous sequence targeted for editing, the targeting guide RNA sequence (if applicable) and how the editor was applied.*
Authentication	*Describe any authentication procedures for each seed stock used or novel genotype generated. Describe any experiments used to assess the effect of a mutation and, where applicable, how potential secondary effects (e.g. second site T-DNA insertions, mosiacism, off-target gene editing) were examined.*

Flow Cytometry

Plots

Confirm that:

- [x] The axis labels state the marker and fluorochrome used (e.g. CD4-FITC).
- [x] The axis scales are clearly visible. Include numbers along axes only for bottom left plot of group (a 'group' is an analysis of identical markers).
- [x] All plots are contour plots with outliers or pseudocolor plots.
- [x] A numerical value for number of cells or percentage (with statistics) is provided.

Methodology

Sample preparation	Mouse (BALF, Lungs):

BALF cells were collected by gently washing the lungs with Hank's balanced salt solution containing 10 vol% FCS using an 18G plastic cannula and a 1 mL syringe. After collecting the BALF cells, the lungs were removed and minced with scissors in Hank's balanced salt solution containing 10 vol% FCS. The minced lungs were incubated with Liberase (#5401127001, Roche, Basel, Switzerland; final concentration: 50 μg·mL–1) and DNase I (#10104159001, Roche; final concentration: 1 μg·mL–1) for 45 min, at 37 °C. The digested lungs were further dissociated with gentleMACS™ (Miltenyi Biotec, Bergisch Gladbach, Germany), and the isolated cells were collected by passing through a 40-μm cell strainer. After lysing the red blood cells with ACK lysing buffer, immune cells were suspended in 30 vol% Percoll PLUS (#17-5445-01, GE Healthcare, Chicago, IL, USA) and centrifuged at 800×g, 24°C for 30 min, to remove epithelial cells. The resulting pellets were used for subsequent analysis after passing through a 37-μm filter. For epithelial cells and fibroblasts, the process of cell separation using Percoll was not performed.

Human (Peripheral blood):
Peripheral blood mononuclear cells were isolated from the whole blood using a BD Vacutainer CPT Mononuclear Cell Preparation Tube (BD Biosciences), suspended in CELLBANKER 1 plus (TaKaRa), and stored at –80 °C until analysis.

Instrument

Data were taken by FACSAria IIIu (BD FACS Diva Software Version 9.0.1) or FACSCanto II flow cytometer (BD FACS Diva Software Version 8.0.2) (BD Biosciences).

Software

Data were analysed by FlowJo software (v10.8.1) (BD Biosciences).

Cell population abundance

Gating strategies was confirmed by backgating, and post-sort fractions were analyzed for checking the purity in some experiments.

Gating strategy

Gating strategies are reported in method section and Extended Data Fig.5b, 5e, 8a, 8c, 9, and 10.

FSC-A/SSC-A gates were used to exclude debris.
FSC-H/FSC-W and SSC-H/SSC-W, or FSC-A/FSC-H gates were used to exclude doublet cells.

For analysis:
ILC2s were defined by CD45+lineage- (CD3ε, CD4, CD8α, CD11c, FcεRIα, NK1.1, CD19, TER119, F4/80, Ly-6G and Ly-6C) Thy1 +ST2+KLRG1+.
ST2-KLRG1- cells (ILC3-like cells) were defined by CD45+lineage- (CD3ε, CD4, CD8α, CD11c, FcεRIα, NK1.1, CD19, TER119, F4/80, Ly-6G and Ly-6C) Thy1+ST2-KLRG1-.
Siglecf+CD11c+Alveolar macrophages were defined by CD45+Gr-1-F4/80+Siglecf+CD11+.
SiglecflowCD11c+ macrophages were defined by CD45+Gr-1-F4/80+SiglecflowCD11+.
Epithelial cells were defined by TER119-CD31-CD45-EpCAM+PDGFRα-.
Fibroblasts were defined by TER119-CD31-CD45-EpCAM-PDGFRα+.

For sorting of mouse ILC2s, fibroblasts, and epithelial cells:
ILC2s were defined by CD45+lineage-Thy1+ST2+KLRG1+.
Fibroblasts were defined by TER119-CD31-CD45-EpCAM-PDGFRα+.
Epithelial cells were defined by TER119-CD31-CD45-EpCAM+PDGFRα-.

For sorting of human ILC2s:
ILC2s were defined by Lineage-CD45+CD161+CRTH2+CD127+.

☒ Tick this box to confirm that a figure exemplifying the gating strategy is provided in the Supplementary Information.
